# Evaluation of Microsatellite Instability Molecular Analysis versus Immuno-Histochemical Interpretation in Malignant Neoplasms with Different Localizations

**DOI:** 10.3390/cancers15020353

**Published:** 2023-01-05

**Authors:** Maria Sfakianaki, Maria Tzardi, Konstantina Tsantaki, Chara Koutoulaki, Ippokratis Messaritakis, Galateia Datseri, Eleni Moustou, Dimitrios Mavroudis, John Souglakos

**Affiliations:** 1 Laboratory of Translational Oncology, School of Medicine, University of Crete, 71003 Heraklion, Greece; 2 Department of Pathology, University General Hospital of Heraklion, 71110 Heraklion, Greece; 3 Department of Medical Oncology, University General Hospital of Heraklion, 71500 Iraklio, Greece

**Keywords:** microsatellite instability, colorectal cancer, MMR proteins, fragment analysis, *MSH2*, *MLH1*, *MSH6*, *PMS2*, dMMR, pMMR, MSI-High

## Abstract

**Simple Summary:**

Mismatch repair (MMR) system deficiency results in increased mutation rates with consequent microsatellite instability (MSI) and susceptibility to carcinogenesis. Clinically, testing MSI status contributes not only to early Lynch syndrome detection, which is associated with an increased risk of various cancers, but also to predicting the biomarkers of response to immune checkpoint inhibitors. In addition, it works prognostically because patients with the MSI phenotype or deficient MMR system (MSI-H or dMMR) characteristics show improved overall survival compared to patients with microsatellite stability or a proficient MMR system (MSS or pMMR). Here, we compare the two methods for MSI testing and outline the pros and cons of both methodologies, as well as examine their sensitivity, complementation, and degree of concordance to clarify to clinicians the ultimate methodology for MSI testing for different cancer types. Both methods are generally known to produce false negative results under certain circumstances, even in technically ideal situations. It is recommended that both methods ought to be established for determining MSI-H and dMMR status, respectively, for all cancer types as a first-line screening test, regarding the substantial agreement of the two methods in the present study.

**Abstract:**

MMR gene germline mutations are considered a major genetic disorder in patients with hereditary nonpolyposis colon cancer (HNPCC) or Lynch syndrome; A total of 15% of sporadic colon carcinomas are MSI-High. MSI has also been observed in other cancers, such as endometrial, gastric, and ovarian cancer. The aim of the current study was to correlate and outline the optimal method between the molecular testing of the instability of microsatellite DNA regions (MSI status) and the loss of protein expression by immunehistochemistry (MMR). A total of 242 paraffin-embedded tissues from gastrointestinal, gynecological, genitourinary, lung, breast, and unknown primary cancer patients were analyzed for the expression of *MLH1*/*MSH2*/*MSH6*/*PMS2* by immunohistochemistry, as well as for the molecular analysis of MSI status using PCR-based molecular fragment analysis. A total of 29 MSI-High patients were detected molecularly, while 23 patients were detected by immunohistochemistry, with rates that are comparable according to the literature. Based on the agreement coefficient of the two methods, a substantial agreement emerged (Kappa = 0.675 with standard error = 0.081, *p* < 0.001). Despite the substantial agreement, both methods ought to be established to determine MSI-H/dMMR status in all cancer types as a first-line screening test.

## 1. Introduction

Microsatellites are regions of DNA consisting of continuous repeats of 1–6 nucleotides; these can distribute throughout the human genome and have a higher mutation rate than other areas of DNA, leading to high genetic diversity [1,2]. The mismatch repair (MMR) pathway is a critical, multimechanism of a cell to repair DNA errors during normal DNA replication and recombination [3]. MMR system deficiency results in a greatly increased mutation rate with consequent microsatellite instability (MSI) and susceptibility to carcinogenesis [4] or abnormal methylation of the promoter region of the *MLH1* gene [5]. The main protein complexes involved in the MMR system are MutSa (*MSH2*/*MSH6*) and MutLa (*MLH1*/*PMS2*) [6,7]. The most common mutations in the repair system occur in the *MSH2 MLH1*, *MSH6* and *PMS2* genes, while the number of recorded *MSH3* and *PMS1* gene mutations is lower [8,9].

Clinically, clarifying MSI status contributes to the early detection of Lynch syndrome, which is an inherited cancer syndrome caused by a germline mutation in one of the several genes involved in the MMR pathway, including *MLH1*, *MSH2*, *MSH6*, and *PMS2.* Lynch syndrome is associated with an increased risk of various cancers, such as colorectal, endometrial, gastric, ovarian, small bowel, and hepatobiliary, as well as urothelial cancers, which may occur synchronously or metachronously [10].

In addition, it works prognostically since patients with the MSI-High phenotype or a deficient MMR system (MSI-H or dMMR) characteristics show improved overall survival compared to patients with microsatellite stability or a proficient MMR system (MSS or pMMR) [11]. Therapeutically, it is a powerful tool since MSI-H tumors respond to immunotherapy (e.g., PD-1 and PDL-1 checkpoint inhibitors) [1,12]. To date, the MSI status of a tumor is the only robust predictive biomarker of response to immune checkpoint inhibitors [13]. Last but not least, the benefits of genetic testing also extend to at-risk family members, who may use the information as a primary means to prevent cancer [14].

Several studies have indicated that MSI testing via PCR-based molecular fragment analysis or immunehistochemistry (IHC) are both highly effective and equally informative established methodologies [5,15,16]. Here, we compare both methods of MSI testing and outline the pros and cons of these methodologies, as well as examine their sensitivity, complementation, and degree of concordance to clarify to clinicians the ultimate methodology for MSI testing in different cancer types. The present study is a comparison of the main methodologies, the molecular testing of the instability of the microsatellite DNA regions (MSI status), and the loss of protein expression by immunohistochemistry (MMR status).

## 2. Materials and Methods

From July 2014 to October 2020, 242 newly diagnosed and histologically documented tumors in patients with 19 different solid tumors arising from tissues that do not include fluid areas were enrolled in the study and diagnosed at the Department of Medical Oncology, University Hospital of Heraklion (Greece). Relevant clinical information was obtained from the medical oncology examination request forms. The median age of the patients was 64 years (range, 19–89 years), and 131 (54.1%) of them were males and 111 (45.9%) females; a total of 90 (37.2%) had metastatic disease, 160 (66.1%) had colorectal cancer, 9 (3.9%) had endometrial cancer, 14 (5.7%) had gastric cancer, 13 (5.3%) had pancreatic cancer, 1 (0.4%) had anal cancer, 3 (1.2%) had duodenal cancer, 3 (1.2%) had hepatocellular carcinoma, 2 (0.8%) had malignant mesothelioma, 7 (2.9%) had biliary cancer, 2 (0.8%) had small intestine cancer, 4 (1.6%) had breast cancer, 2 (0.8%) had ovarian cancer, 6 (2.5%) had esophageal cancer, 4 (1.6%) had unknown primary cancer, 1 (0.4%) had bladder cancer, 5 (2%) had cervical cancer, 4 (1.6%) had lung cancer, 1 (0.4%) had sarcoma, and 1 (0.4%) had brain cancer. The study was approved by the Ethics Committee/Institutional Review Board of the University Hospital of Heraklion (Greece) (Number: 7302/19-8-2009), and all patients signed written informed consent for their participation in the study.

Formalin-fixed, paraffin-embedded (FFPE) tissues (5 μm-thick) from all patients with different neoplasms and with histologically confirmed stages were collected and analyzed. In addition, we collected and analyzed the paired normal tissue sections (3 μm-thick) from all participants enrolled in the study, selected by an experienced pathologist. Microdissection or macrodissection of the cancer cells from the FFPE sections and DNA extraction were performed as previously described [17,18].

### 2.1. Immunohistochemistry (IHC) Testing

**Figure 1:**
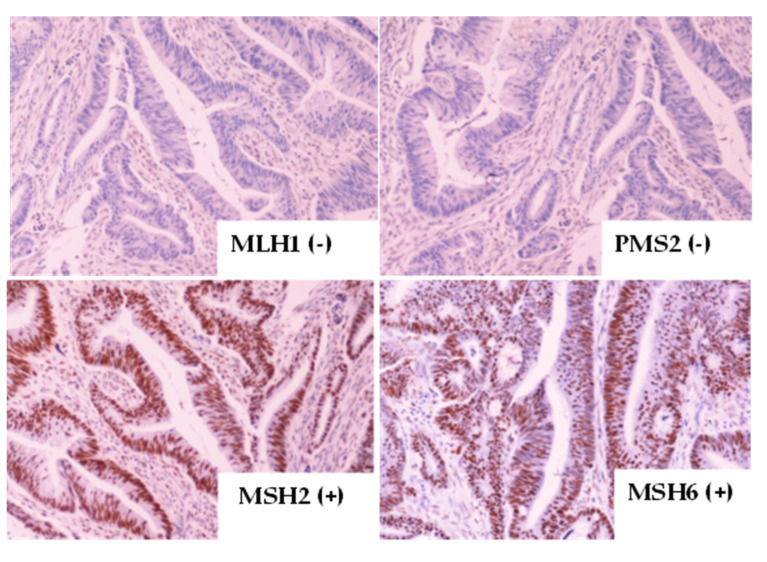
Colorectal cancer tissue with *MLH1* and *PMS2* negative expression (absence of nuclear staining) and *MSH2* and *MSH6* positive expression (presence of nuclear staining).

The immuno-histochemical staining of *MLH1*/*MSH2*/*MSH6* and *PMS2* MMR proteins was performed in 230 patients by Ultravision Quanto Detection System HRP DAB (Thermo Scientific, Waltham, MA, USA) and the DAB Quanto (Thermo Scientific, Cheshire WA7 1TA, UK). Briefly, 3 μm sections cut from the formalin-fixed, paraffin-embedded (FFPE) tumor tissue blocks were stained with monoclonal antibodies to MLH1 (Clone: ES05 Agilent Dako, Santa Clara, CA, USA), MSH2 (Clone FE 11 Agilent Dako), MSH6 (Clone: EP49 Agilent Dako), and PMS2 (Clone: EP51 Agilent Dako). The evaluation of the immuno-histochemical expression of the four proteins was performed by a specialized pathologist in the Department of Pathology, University General Hospital of Heraklion. MMR protein immuno-histochemical expression was considered positive when located in the nucleus. In all cases, a positive control of colorectal carcinoma MMR-stable, confirmed by PCR, as well in some cases, an internal positive control (lymphocytes, epithelial cells of colonic crypts), was used. Nuclear staining in all cell types was considered a positive stain. As negative control slides were used from FFPE colorectal cancer tissues, MMR-stable with the primary antibody was omitted. Additionally, an internal positive control (lymphocytes and colonic crypts) was also used. FFPE tumor sections with no primary antibodies were used as the negative control. pMMR protein expression is highlighted by unequivocal nuclear staining in the tumor cells, as previously described [19] when the internal positive controls (lymphocytes, fibroblasts, or normal enterocytes in the vicinity of the tumor) showed positive nuclear staining. In contrast, the tumor was categorized as having dMMR when the nuclear staining of tumor cells was absent (Figure 1) for one or more of the MMR proteins, despite immunoreactivity in the internal positive controls. When internal controls failed, the samples were excluded from the study.

### 2.2. PCR with Fragment Analysis

For MSI fragment analysis, fluorescent labeled DNA fragments from each tumor and the normal tissues, separated by capillary electrophoresis, were analyzed. Five mononucleotide markers (NR-21, BAT-26, BAT-25, NR-24, and MONO-27) were simultaneously used and sized by comparison to an internal standard using the Promega MSI Analysis System (Version 1.2; Madison, WI, USA), according to the manufacturer’s instructions. Two pentanucleotide markers (PentaC, PentaD) were also used to detect potential contamination. The MSI status was defined in accordance with the Bethesda guidelines [20,21]. Detection of the amplified loci used the ABI Prism 3100 genetic Analyzer (Applied Biosystems, Foster City, CA, USA) with Data Collection Software, Version 4.1 ((Applied Biosystems, Foster City, CA, USA).

**Figure 2:**
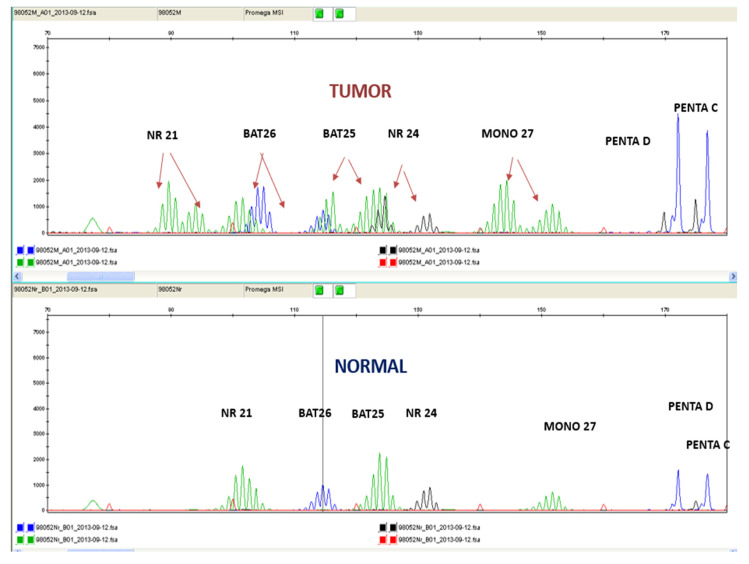
Electropherogram using the pentaplex panel comparing normal tissue samples (bottom panel) with tumor tissue samples (top panel) of the same patient. The additional peaks present on the top panel are a result of MSI. Red arrows indicate microsatellite instability.

Samples with instability were defined as any change in length of a detection loci of more than two base pairs when compared to the same marker in the normal sample. A tumor was determined as MSI-H when two or more of the five loci were unstable. When one marker was unstable, it was designated as MSI-Low (MSI-L). Tumors with no detectable unstable loci were MSI-Stable (MSS) (Figure 2).

## 3. Results

### 3.1. MSI and MMR Status Analysis of Various Malignant Neoplasms

Patients’ characteristics are listed in Table 1 and Table S1. The *BRAF* mutations were tested in only 110 (45.5%) of the colorectal cancer patients, and 11 (4.9%) were *V600E* mutants. Within the cohort of 242 patients, 12 patients were not tested for their MMR system status by IHC due to insufficient tumor specimens. Only one patient was not tested for MSI status (molecularly) because of a low DNA quantity. Of the tested patients, a total of 30 (12.4%) and 21 (8.7%) were of MSI-H and dMMR status, respectively (Table 1 and Table S1). In brief, MSI-H was detected in 15.6% (25 out of 135) of the colon cancers, in 44.4% (4 out of 9) of the endometrial cancers, in 7.1% (1 out of 13) of the gastric cancers, and in 6.9% (4 out of 58) of the other solid tumors.

### 3.2. MSI-H vs. dMMR Status in Different Solid Tumors

**Figure 3:**
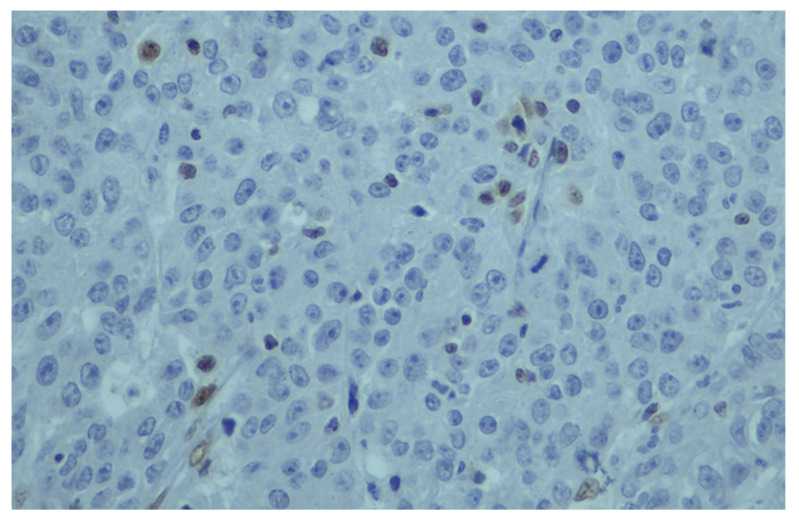
Immuno-histochemical staining of *MLH1* in a few tumor cells which contradicts the result of fragment analysis.

**Figure 4:**
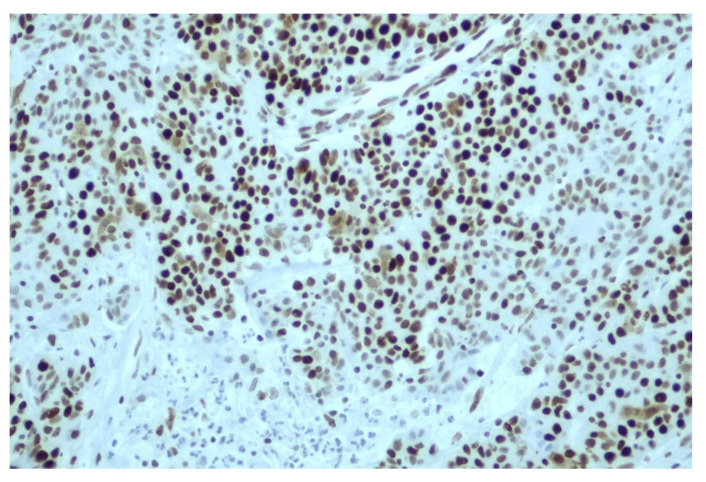
Immuno-histochemical staining of *MSH2* in concordance with the results of the fragment analysis.

The concordance between MSI-H and dMMR status was moderate, with 81% of dMMR IHC showing MSI-H status; in contrast, 63% of cases with MSI-H were dMMR as per IHC (Table 2a). The agreement between the two methods was calculated by Cohen’s kappa coefficient, which is an important statistical test in determining the inter-rater reliability between the two methods. A substantial agreement resulted from the inter-rater analysis between the two methods (Kappa = 0.675 with standard error = 0.081, *p* < 0.001). A discordance between the MSI and MMR tests was observed in 15 patients; in brief, the PCR fragment analysis revealed 10 MSI-H patients. Figure 3 and Figure 4 demonstrate the two different patterns of protein IHC for patients with a pMMR status. Additionally, Figure 3 present a positive *MLH1* staining as a result of MSI-H by the fragment analysis method. On the other hand, only five patients with a dMMR status were MSS. The sensitivity of MSI and IHC testing was 90.5% and 65.4%, respectively, whereas, the specificity was 96.2% and 98%, respectively.

Colorectal cancer presented a higher frequency of MSI with 14.46% and 9.93% by PCR fragment analysis and IHC, respectively. Additionally, nine endometrial tumors were assessed for the MSI analysis and IHC analysis of the MMR system, whereas, interestingly, two (22.2%) and three (33.3%) cases resulted in MSI-H and dMMR, respectively (Table 2b). Although dMMR in endometrial cancer (33.3%) was found to be increased, it was not statistically significant, possibly due to the small number of samples (n = 9). On the other hand, none of the pancreatic cancers (n = 13) showed MSI-H nor dMMR status in the current study (Table 2b).

Statistical analysis found a higher frequency in patients with tumor grade II with respect to MSI-H status and the dMMR system. Both these correlations were statistically significant; *p* = 0.017 for MSI-H and *p*= 0.008 for dMMR status (Table 3a,b). In addition, both MSI-H status (85.7%, *p* = 0.005) and dMMR (85%, *p* = 0.056) status correlated significantly with nonmetastatic patients (Table 3c).

## 4. Discussion

In the current study, molecular or immuno-histochemical analysis for microsatellite instability across 19 various tumors resulted in a higher prevalence rate of endometrial (33%) followed by colon (14.5%) cancer. The epidemiology of MSI across colorectal cancer has stated that MSI-H/dMMR status occurs more frequently in stage II (~20%) compared to stages III (~12%) and IV (~4%), and this is in the same line of evidence with previous studies [22,23]. A previous meta-analysis reported the highest MSI-H and dMMR prevalence in endometrial cancer (26%) across solid tumors [23]. Similarly, another study based on MSI detection among gynecological malignancies showed that endometrial cancer represents the highest percentage, with approximately 30%, followed by ovarian carcinoma accounting for 10–15% [24]. In addition, several studies that investigated MSI-H or dMMR status found these in 2–12% of metastatic prostate cancers and showed the clinical activity of pembrolizumab in such cases [25,26]. Moreover, the MSI frequency in gastric cancer is estimated to be 10–20% [27]. Similarly, in the present study, the prevalence of MSI-H/dMMR status in gastric cancer was 7%.

Of notice in the current study is the fact that none of the pancreatic cancer patients were detected with MSI-H/dMMR status, which is a rate consistent with the updated guidelines of the National Comprehensive Cancer Network (NCCN) [28,29]. However, two recent studies present conflicting data, with 12–17% MSI-H for pancreatic patients [30,31]. No matter the MSI-H/dMMR status of the pancreatic patients, it is worth noting that, in a pivotal trial of pembrolizumab, 83% of the patients with dMMR pancreatic cancer reported a great response [28].

MSI testing was a major shift for healthcare systems since all patients with colorectal cancer should be tested for the detection of Lynch syndrome [32]; however, it is also important to use this for the decision of immune checkpoint inhibitor therapy administration in advanced stages for several tumor types [33]. Furthermore, early diagnosis of MSI/MMR status is valuable for the omission of adjuvant chemotherapy in stage II colon cancer patients. [34]. Nowadays, the analysis of the MMR system proteins and the MSI status of many solid tumors is established as a matter of routine [12].

Limited data exist for a comparison between MSI testing and MMR IHC in the overall detection of MSI-H/dMMR in different tumor types. Our study sought to thoroughly compare both diagnostic assays to determine their sensitivity and degree of concordance and clarify to clinicians the ultimate methodology for MSI testing in different cancer types. The K value indicated a substantial agreement between the two methodologies in a large cohort with several solid tumors. The appreciable rate of concordance in our study (Kappa = 0.675) is comparable to or lower than previous studies reporting on the populations of colon cancers [35,36,37]. The discordant results in our cohort constituted 10 MSI-H in colorectal cancer cases which were diagnosed as pMMR by IHC, and it was observed, interestingly, that they showed protein staining only in a very small percentage of the cells (or else, an unusual pattern of staining). A logical and potential explanation after the repeated evaluation of the discordant cases by the expert pathologist was the small percentage of the immunostained cells. When interpreting MMR, IHC loss of protein expression is defined as the complete absence of nuclear staining within the tumors [5]. Likewise, when more than 10% of the tumor cells showed a lack of or reduced expression of these markers, the tumor was regarded as negative for protein expression [38]. Previous studies have evidenced that an explanatory, semiquantitative scoring system based on the immunostaining percentage of tumor cells is reproducible and is beneficial over standard scoring methods based on predetermined cut-off scores [39]. Consequently, the results of our study recommend that there is a need for awareness of the evaluation of a lower percent positivity cut-off.

It is also important to state the rationale that about 5% of colorectal cancers with a pMMR status may not absolutely exclude the possibility of an MSI-H status. A logical explanation for the discordance between the IHC and PCR fragment analyses includes an unevaluated MMR gene product as the cause of the defect or that one of the MMR genes tested is expressed but has suboptimal function and/or is nonfunctional, possibly due to a missense mutation [5,40]. In these cases, the molecular method of MSI can help to determine whether there are true functional MMR proteins through these mutations.

MSI molecular detection of specific microsatellite repeats and IHC detection of the loss or presence of MMR proteins measure two analogous but diverse parameters. The determination of the suitability of one methodology or another as a primary screening test is not clear. IHC is a more familiar examination and is commonly used in a routine cellular pathology laboratory in the developed world. The outstanding advantage of IHC over PCR fragment analysis is the identification of the MMR gene product, and this assists with mutational confirmation in the future. MMR IHC detection (rather than MSI testing) requires normal tissue to be cotested to define the presence of MSI when the only available tissue for testing is the tumor.

Nevertheless, in laboratories with access to molecular pathology services, PCR, followed by fragment analysis, has potential pros over MMR IHC [35,37]. In our laboratory, MSI testing makes use of various combinations of microsatellite markers, except the Promega panel, as this results in variations in sensitivity and specificity. Furthermore, only PCR fragment analysis is employed for MSI testing in the case of diagnostic endoscopic biopsy specimens [35].

A limitation of the current study is the small sampling size of some of the different cancer types, such as ovarian, breast, small intestine, and lung. Consequently, a future perspective for our laboratory would be the enrichment of more samples for MSI testing from collaborating hospitals to provide complementary information.

When putting aside the advantages and disadvantages reported above, both methods are generally known to produce false negative results under certain circumstances, even in technically ideal situations. In summary, both methods ought to be established for determining MSI-H/dMMR status in all cancer types as a first-line screening test, regarding the substantial agreement of the two methods in the present study. However, caution is advised for the rare cases with a small percentage of scattered cells with positivity expression. In that case, PCR, followed by fragment analysis, would be used as a ground-through method. Briefly, having both methods available is desirable to enable the interpretation of difficult or extraordinary cases and design this important molecular subtype for several types of cancer with maximum accuracy.

## 5. Conclusions

The current study provides a comparison of the main methodologies for the molecular testing of the instability of the microsatellite DNA regions (MSI status) and the loss of protein expression by immunohistochemistry (MMR). Molecular or immuno-histochemical testing for MSI-H or dMMR across 19 tumor types in the current study resulted in a higher prevalence rate for endometrial followed by colon cancer. A total of 29 patients with MSI-H were detected molecularly, while 23 patients were detected immuno-histochemically, with rates comparable to the literature. Based on the agreement coefficient of the two methods, a substantial agreement emerged according to the Kappa value analysis. The discordant results in our cohort constitute 10 MSI-H in the CRC cases which were diagnosed pMMR by IHC, and it was observed, interestingly, that they showed protein staining only for a very small percentage of the cells (or else, an unusual pattern of staining). To conclude, both methods ought to be established to determine MSI-H/dMMR status for all cancer types as a first-line screening test, regarding the substantial agreement of the two methods.

## Figures and Tables

**Table 1:** Clinical characteristics and pathological features.

Feature	N	%
	242	
Median Age (Range)	64 (19–89)	
<64 years	158	65.3
≥64	79	32.6
Gender		
Female	111	4.9
Male	131	54.1
Patients		
Adjuvant	142	58.7
Metastatic	90	37.2
Unknown	10	4.1
*BRAF^V600E^* status		
WT	99	40.9
Mutant	11	4.5
Not Done	132	54.5
MMR Status		
dMMR	21	8.7
pMMR	209	86.4
Not Done	12	5.7
MSI Status		
MSI-High	29	12
MSI-Stable	212	87.6
Failed	1	0.4

**Table 2:** MSI by PCR_Fragment Analysis vs. MSI by immunohistochemistry crosstabulation.

		MMR		
		dMMR	pMMR *p* Value	
MSI Total	High	18 (64.3%)	10 (35.7%)	
	Stable	5 (2.5%) 23	197 (97.5%) > 0.001 207	
(**a**) Analysis of MSI Status in various malignant neoplasms by PCR-Fragment Analysis				
**Neoplasm**	**N**	**%**	**MSI-H Status**	**%**
Colorectal	160	66.1	23	14.46
Stomach	14	5.7	1	7.1
Pancreas	13	5.3	0	0
Endometrium	9	3.7	2	22.2
Biliary	7	2.9	0	0
Esophageal	6	2.5	0	0
Cervix	5	2.0	0	0
Breast	4	1.6	1	25.0
Unknown primary	4	1.6	0	0
Lung	4	1.6	1	25.0
Liver	3	1.2	0	0
Duodenum	3	1.2	0	0
Mesothelioma	2	0.8	0	0
Ovarian	2	0.8	0	0
Small Intestine	2	0.8	0	0
Anal	1	0.4	0	0
Bladder	1	0.4	0	0
Sarkoma	1	0.4	0	0
Brain	1	0.4	0	0
Total	242	100	28	11.57
(**b**) Analysis of MMR Status in various malignant neoplasms by ICH.				
Colorectal Stomach	151 13	65.7 5.7	15 1	9.93 7.69
Pancreas Endometrial	13 9	5.7 3.9	3	0 33.0
Biliary	7	2.9	0	0
Esophageal	6	2.5	0	0
Cervix	5	2.0	0	0
Lung	4	1.7	1	25.0
Liver	3	1.3	0	0
Duodenum	3	1.2	1	33.3
Breast	3	1.3	0	0
Unknown primary	3	1.3	0	0
Mesothelioma	2	0.9	0	0
Small Intestine	2	0.9	0	0
Ovarian	2	0.9	0	0
Bladder	1	0.4	0	0
Sarcoma	1	0.4	0	0
Anal	1	0.4	0	0
Brain	1	0.4	0	0
Total	242	100	21	8.67

**Table 3:** (**a**) Description of patients number according to their disease stage. (**b**) Correlation of tumor stage with MSI and MMR status. (**c**) Correlation of MSI or MMR status with metastatic or non-metastatic patients.

(a)					
Stage	N	%	MSI-H (%)		dMMR (%)
IA	25	10.2	4 (15.4)		3 (15)
II	4	1.6	1 (25)		1 (25)
IIA	36	14.8	9 (25)		7 (20.6)
IIB	7	2.9	1 (14.3)		1 (14.3)
III	6	2.5	1 (20)		0
IIIA	3	1.2	1 (33.3)		0
IIIB	28	11.5	1 (3.6)		0
IIIC	27	11.1	2 (7.7)		2 (7.7)
IV	88	36.1	6 (6.8)		5 (5.8)
IVA	4	1.6	0		0
Unknown	16	6.6	0		0
Total	242	100	26		19
(b)					
	**I**	**II**	**III**	**IV**	***p*** **Value**
MSI-High	4 (1.7%)	12 (5.2%)	7 (3.0%)	5 (2.2%)	0.017
MSS Total	21 (9.1%) 25	40 (17.2%) 52	56 (24.1%) 63	87 (37.5%) 92	
dMMR	4 (1.8%)	9(4.1%)	2 (1.8%)	5 (2.3%)	0.008
pMMR Total	17 (7.7%) 21	39 (17.7%) 48	59 (26.8%) 61	85 (38.6%) 90	
(c)					
	**M0**		**M1**		***p*** **Value**
MSI-High	24 (85.7%)		4 (14.3%)		0.005
MSS Total	118 (57.8%) 142		86 (42.2%) 9 90		
dMMR	16 (80%)		4 (20.0%)		0.056
pMMR	116 (58%)		84 (42.0%)		
Total	132		88		

## Data Availability

All relevant data are within the paper and its Supporting Information files.

## Supplementary Materials

The following supporting information can be downloaded at: https://www.mdpi.com/article/10.3390/cancers15020353/s1, Raw data information.

## Glossary

MLH1: MutL protein homolog 1 gene, MSH2: MutS homolog 2 gene, MSH6: mutS homolog 6 gene, PMS2: PMS1 Homolog 2 gene, MSS Microsatellite Stable, pMMR: proficient mismatch repair pathway, PD-1: programmed cell death protein 1, PDL-1: programmed death-ligand 1.
